# Associations Between Conduct Disorder, Neurodevelopmental Problems and Psychopathic Personality Traits in a Swedish Twin Youth Population

**DOI:** 10.1007/s10862-018-9689-z

**Published:** 2018-06-29

**Authors:** Olof Svensson, Karolina Sörman, Natalie Durbeej, Paul Lichtenstein, Henrik Anckarsäter, Nora Kerekes, Thomas Nilsson

**Affiliations:** 1 National Board of Forensic Medicine, Department of Forensic Psychiatry, 422 49 Gothenburg, Sweden; 2 Department of Clinical Neuroscience, Karolinska Institutet, Stockholm, Sweden; 3 Department of Medical Epidemiology and Biostatistics, Karolinska Institutet, Stockholm, Sweden; 4 Centre for Ethics, Law and Mental Health, University of Gothenburg, Gothenburg, Sweden; 5 Department of Health Sciences, University West, Trollhättan, Sweden

**Keywords:** Conduct disorder, Neurodevelopmental problems, Psychopathic traits, Callous-unemotional, Grandiose-deceitful

## Abstract

Previous research has found a complex relationship between psychopathic traits, neurodevelopmental problems (NDPs), and conduct disorder (CD) in children. This study explores associations between psychopathic traits, assessed with the Child Problematic Traits Inventory—Short Version (CPTI-SV), and CD in children with and without coexisting NDPs (i.e., attention deficit/hyperactivity disorder [ADHD] and autism spectrum disorder [ASD]) in a community-based sample of Swedish twins (*n* = 8762). Findings indicate weak to moderately strong correlations between psychopathic traits and CD, ADHD, and ASD, respectively. Furthermore, in univariable analyses, both psychopathic traits and NDPs displayed significant positive associations with being screened positive for CD, though only the grandiose-deceitful dimension of CPTI-SV and the ADHD domain concentration and attention deficits remained significantly associated with CD in a multivariable regression model. The results are relevant to screening and assessment in child and youth psychiatry, as a grandiose and deceitful interpersonal style may also be a valid sign of children at risk of developing CD.

## Introduction

Accumulating research on the clinical relevance of callous-unemotional (CU) traits in youth has prompted change in the DSM classification system, reflecting the importance of assessing such traits in children. Earlier DSM editions (i.e., DSM-III/IV; APA 1994, 2000) further specified conduct disorder (CD) diagnosis only by the age of onset, differentiating between childhood and adolescent onset. In the DSM-5 this specification has been supplemented with the specifier “with limited prosocial emotions” (LPE), assessing whether individuals demonstrate limited prosocial emotions, including reduced guilt and empathy (APA 2013, p. 470). The LPE specifier operationalizes personality features reflecting “callous-unemotional” (CU) traits. However, in a recent review, Salekin (2017) noted that DSM-5 includes no similar specifiers covering additional dimensions of psychopathic traits in children, despite mounting research indicating that both grandiose-manipulative (also conceptualized as grandiose-deceitful, GD) and daring-impulsive (also conceptualized as impulsivity/need for stimulation, INS) traits can be reliably assessed and exhibit theoretically relevant outcomes in youth. According to Salekin (2017), grandiose-manipulative traits are related to both increased aggressive behavior and decreased prosocial behavior, with some research indicating that this relationship is even stronger with GD traits than CU traits. Daring-impulsive traits, in contrast, appear to be more related to educational difficulties and general risk-taking behaviors, but have also been associated with elevated conduct problems (Salekin 2016).

Research has so far narrowly focused on associations between CU traits and CD. Although this has improved our knowledge, we need additional research covering the broader concept of psychopathy, including all its underlying dimensions (Salekin 2016). Other topics also warrant further research, such as interactions between CD and childhood-onset neurodevelopmental problems (NDPs) that could affect developmental pathways to aggressive antisocial behavior in adults. Previous research suggests that subgroups of youths with neurocognitive dysfunctions (e.g., deficient decision-making) also display CD (Blair et al. 2014). Several studies have demonstrated considerable overlap between the presence of CD and neurodevelopmental problems, such as attention deficit hyperactivity disorder (ADHD) and autism spectrum disorder (ASD) (e.g., Kerekes et al. 2014; Lundström et al. 2011).

In reviewing the adult phenotype of antisocial personality disorder (ASPD), Hofvander et al. (2009) demonstrated that one developmental trajectory of this behavioral condition could be regarded as progression from childhood ADHD to subsequent oppositional defiant disorder and eventual CD. According to Hofvander et al. (2009), about half of all children on this trajectory would meet ASPD criteria as adults. It has also been suggested that varying degrees of CU and psychopathic traits can be present in individuals with ASD, although the data suggest that these tendencies likely express different affective/informational processes (Jones et al. 2010). Another theory is that such a combination of traits could reflect a “double hit”, that is CU traits being an additional impairment adding further dysfunction to ASDs (Rogers et al. 2006). A recent study of genetic and environmental influences demonstrated that ASDs and elevated CU traits, while superficially similar, are etiologically independent (O’Nions et al. 2015). In summary, the relationship between NDPs (i.e., ADHD and ASD), CD, and psychopathic personality traits (CU traits so far have been the primary research focus) is complex and requires further study.

## Aims

The current study investigates the degree to which psychopathic traits are associated with CD beyond NDPs, specifically:

1. describing the distribution of psychopathic traits, assessed with the Child Problematic Traits Inventory—Short Version (CPTI-SV), in a Swedish general population-based sample of twins (age 9 or 12);
2. investigating zero-order correlations between psychopathic traits, degree of CD symptoms, and two NDPs (i.e., ADHD and ASD); and
3. investigating associations between psychopathic traits and CD, taking two NDPs (i.e., ADHD and ASD) into account, by means of logistic regressions.

## Method

Participants were selected from the ongoing Child and Adolescent Twin Study in Sweden (CATSS), a nationwide longitudinal study of twins from the age of 9 years whose inclusion started in 2004 (see Anckarsäter et al. 2011, for a detailed presentation of CATSS). The overall response rate for this study has been high, with an 80% inclusion rate as of the beginning of 2010. Participants were 9- and 12-year-old twins whose parents completed all CPTI-SV items (Colins et al. 2014) and the Autism—Tics, AD/HD and other Comorbidities (A-TAC) inventory (Larson 2013). From the total dataset (*N* = 22,202) a subsample of 8762 individuals met these criteria, of whom 4453 (50.8%) were boys and 4309 (49.2%) were girls. In this study sample, 4185 (47.8%) were 9 years old and 4577 (52.2%) were 12 years old at baseline. Zygosity was distributed as follows: 29% (2545) monozygotic (MZ), 69.2% (6063) dizygotic (DZ), and 1.8% (154) unknown zygosity twins. The exact numbers of individuals included in specific analyses differ slightly from one another (see Table 3), due to missing data in certain A-TAC modules.

## Instruments

### The Child Problematic Traits Inventory—Short Version (CPTI-SV)

CPTI-SV is a 12-item short form of the original 28-item CPTI, developed to provide a convenient measure of three problematic trait dimensions in children (Colins et al. 2014). These dimensions (or subscales) mirror the adult three-factor model of psychopathy: *grandiose-deceitful* (GD, four items), *callous-unemotional* (CU, four items), and *impulsive-need for stimulation* (INS, four items). The CPTI-SV items are scored on four-point Likert scales ranging from 1 (“does not apply at all”) to 4 (“applies very well”) by an adult who knows the child/adolescent well (i.e., parent or a teacher). The maximum score is 16 points on each subscale and 48 points for the entire CPTI-SV. The three-factor structure of the original CPTI has been demonstrated to be robust for use with children as young as three years old (Colins et al. 2014) and to have generally good psychometric properties (Colins et al. 2014, 2016; Sörman et al. 2017). Specifically, CPTI-SV has good internal consistency for the total scale (α = .79) and for the GD (α = .70) and CU (α = .77) subscales; the INS subscale deviated slightly from this pattern, having somewhat lower internal consistency (α = .67).

### The Autism—Tics, AD/HD and Other Comorbidities Inventory (A-TAC)

A-TAC, a comprehensive inventory for assessing autism and its potential comorbidities, is administered to a parent or other collateral source (Larson 2013). The 17-item ASD module comprises three domains: *language* (six items), *social interaction* (six items), and *flexibility* (five items). Each item is scored on a three-point Likert scale ranging from 0 (= “no”), through 0.5 (= “to some extent”), to 1 (= “yes”). The 18-item ADHD module comprises two nine-item domains: *concentration and attention* and *impulsiveness and activity*. The five-item CD module comprises these items: “Does he/she often lie or cheat?”; “Has he/she ever engaged in shoplifting?”; “Has he/she ever deliberately been physically cruel to anybody?”; “Does he/she often get into fights?”; and “Does he/she steal things at home or outside home?” Depending on the purpose of the assessment, previously validated low or high thresholds can be used. A low threshold is commonly used for broad screening and a more restrictive, higher threshold for narrower screening, for example, to identify an individual with a probable diagnosis. In this study, the high threshold was used to distinguish participants with clinically elevated levels of CD from the general population of twins. In our sample we used a threshold ≥2, which has an AUC of 0.95, a sensitivity of 0.55, and a specificity of 0.98 (Kerekes et al. 2014), resulting in 69 participants of 8759 (i.e., 0.8%) being screened positive for CD.

### Data Analyses

Analyses were conducted in three consecutive steps using SPSS version 20:

1. Tests of normality (Kolmogorov-Smirnov and Shapiro-Wilk tests) were conducted to investigate whether the data were normally distributed. Results indicated that data were non-normally distributed, so non-parametric zero-order correlations were explored.
2. Spearman’s rho (*r*_s_) correlation coefficients were used to examine associations between: (a) CPTI-SV total scores and scores on the A-TAC inventory (i.e., CD, ADHD, and ASD modules), and (b) CPTI-SV subscale scores and scores on the A-TAC modules for the entire study population and for subgroups (i.e., boys and girls as well as 9- and 12-year-olds, separately).
3. Univariable and multivariable logistic regressions were performed using generalized estimated equations (GEEs) to control for dependence within twins, to investigate the associations between the dependent variable CD and the independent variables CPTI-SV scores (i.e., total and subscale scores) and NDPs (i.e., ADHD and ASD as measured by A-TAC). The presence of CD (i.e., being screened positive) was defined using the high threshold (≥2) on the A-TAC inventory CD module, indicating a high probability of the diagnostic criteria being met, and then used as the dependent variable, where binary response models were fitted to the data. All continuous predictor variables (i.e., CPTI-SV or its subscales and A-TAC scores for the ADHD and ASD modules or their domains) were inserted into the model as covariates, while the categorical variables “age group” and “sex” were inserted as cofactors, and their main effects were analyzed in both univariable and multivariable models. The univariable model calculates the odds ratio (OR) of being screened positive for CD based on one predictor. The multivariable model calculates the ORs of being screened positive for CD when several predictors (i.e., scores on CPTI-SV coexisting with A-TAC scores for ADHD and ASD) are included in the model.

## Results

### Distribution of Psychopathic Traits in 9- and 12-Year-Old Boys and Girls

As expected, very few children were rated by their parents as displaying behaviors and personality features indicative of prominent psychopathic traits (see Table 1). In the entire study group (*n* = 8762), the mean CPTI-SV score was 16.41 (SD = 3.87), with a markedly skewed distribution ranging from the lowest possible score of 12 to the maximum score of 48. There were virtually no differences between boys and girls or between 9- and 12-year-old children,^1^ with all groups displaying mean CPTI-SV scores in a similar range. INS scores were higher than those for the other two subscales (i.e., CU and GD); this pattern remained the same, regardless of sex and age. Both GD and CU traits were rare in all subgroups.

**Table 1:** CPTI-SV scores (i.e., total scores and for each subscale) for the whole group, for boys and girls as well as for 9-years and 12 years of age

Variable	Whole group *N* = 8762				Boys *n* = 4453		Girls *n* = 4309		9 years *n* = 4185		12 years *n* = 4577	
	Min.	Max.	Mean	*SD*	Mean	*SD*	Mean	*SD*	Mean	*SD*	Mean	*SD*
CPTI – SV total scale (range; 12–48)	12	48	16.41	3.87	16.7	4.1	16.1	3.6	16.54	3.90	16.29	3.9
GD dimension (range; 4–16)	4	16	4.52	1.19	4.58	1.3	4.45	1.1	4.53	1.20	4.5	1.2
CU dimension (range; 4–16)	4	16	4.59	1.32	4.68	1.4	4.5	1.2	4.58	1.3	4.6	1.3
INS dimension (range; 4–16)	4	16	7.30	2.35	7.44	2.4	7.16	2.3	7.42	2.3	7.19	2.3

*CPTI – SV* Child Problematic Traits Inventory – Short Version

### Correlations Between Psychopathic Personality Traits, Conduct Disorder, and Neurodevelopmental Problems

There were weak to moderately strong zero-order correlations between CPTI-SV-assessed psychopathic traits and scores for the A-TAC module of CD (see Table 2), with the correlations generally being stronger for boys than girls. The strongest of these correlations were found between the CPTI-SV GD subscale and CD (i.e., *r*_s_ = 0.35 for boys and 0.33 for girls). Besides this, there were moderately strong correlations between scores on the A-TAC ADHD module and the CPTI-SV total score (*r*_s_ = 0.49 for boys and 0.44 for girls), and also between the A-TAC ADHD module and the CPTI-SV INS subscale (*r*_s_ = 0.48 for boys and 0.44 for girls). There were also moderately strong correlations between the A-TAC ASD module and the CPTI-SV total score (*r*_s_ = 0.38 for boys and 0.33 for girls). The correlations between the A-TAC ASD module and individual CPTI-SV subscales were weak.

**Table 2:** Spearman rho correlations between CPTI-SV scores (i.e., total scale scores as well as for each dimension) and A-TAC scores on the ADHD, ASD, and CD modules for the whole group as well as for boys and girls

	ADHD^†^	ASD^††^	CD^†††^
CPTI – SV total scale			
Whole group	0.47* (*n* = 8756)	0.36* (*n* = 8759)	0.28* (*n* = 8759)
Boys	0.49* (*n* = 4450)	0.38* (*n* = 4450)	0.31* (*n* = 4451)
Girls	0.44* (*n* = 4306)	0.33* (*n* = 4309)	0.25* (*n* = 4308)
GD dimension			
Whole group	0.26* (*n* = 8756)	0.25* (*n* = 8759)	0.34* (*n* = 8759)
Boys	0.28* (*n* = 4450)	0.26* (*n* = 4450)	0.35* (*n* = 4451)
Girls	0.23* (*n* = 4306)	0.23* (*n* = 4309)	0.33* (*n* = 4308)
CU dimension			
Whole group	0.25* (*n* = 8756)	0.26* (*n* = 8759)	0.24* (*n* = 8759)
Boys	0.27* (*n* = 4450)	0.27* (*n* = 4450)	0.23* (*n* = 4451)
Girls	0.22* (*n* = 4306)	0.24* (*n* = 4309)	0.23* (*n* = 4308)
INS dimension			
Whole group	0.47* (*n* = 8756)	0.32* (*n* = 8759)	0.23* (*n* = 8759)
Boys	0.48* (*n* = 4450)	0.33* (*n* = 4450)	0.26* (*n* = 4451)
Girls	0.44* (*n* = 4306)	0.29* (*n* = 4309)	0.19* (*n* = 4308)

^†^Attention Deficit Hyperactivity Disorder

^††^Autism Spectrum Disorder

^†††^Conduct Disorder

*p ≤ .001

### Association Between Psychopathic Traits and Conduct Disorder in Children

Table 3 summarizes associations between the dependent variable (i.e., being screened positive for CD) and the independent variables (i.e., age, sex, CPTI-SV total and subscale scores, and A-TAC scores for the ADHD and ASD modules, including their respective domains), in univariable and multivariable models. In the univariable analyses, age was not significantly associated with screening positive for CD, though boys were more likely to screen positive than were girls. All other independent variables displayed significant and positive associations with being screened positive for CD when analyzed in univariable models. In the multivariable models, ADHD and the CPTI-SV total score each retained a significant association with CD. When the scores for their subcomponents (i.e., domains and subscales, respectively) were included in the multivariable regression model, the GD subscale of CPTI-SV (OR = 1.61) and the ADHD-related concentration and attention deficits domain (OR = 1.26) significantly increased the risk of screening positive for CD. In this study, the relevant ORs can be considered equivalent to risk ratios, since they are well below 2.5, and the prevalence of being screened positive for CD was under 10% in the study population (Sistrom and Garvan 2004).

**Table 3:** Univariable and multivariable regression analyses using generalized estimated equations with screen positive CD as dependent variable and CPTI-SV (i.e., total scale or dimensions of GD, CU and INS), ADHD (scores on the A-TAC module of ADHD or scores for its A-TAC domains Concentration and Attention and Impulsiveness and & Activity), and ASD (scores on the A-TAC module of ASD or for its A-TAC domains Interaction, Language, and Flexibility) as covariates and age group and sex as cofactors

	n	Min^†^	Max	Mean	*SD*	Univariable model			Multivariable model I			Multivariable model II		
						*p*	OR	95% CI	*p*	OR	95% CI	*p*	OR	95% CI
Age 9 year	4146					0.384	1.27	0.74–2.16						
12 year	4544						1.00							
Sex Boys	4407					0.05	1.72	1.00–2.95	0.846	0.93	0.46—1.90	0.980	0.99	0.48–2.04
Girls	4283						1.00			1.00			1.00	
CPTI – SV total scale	8759	12	48	16.40	3.86	0.001	1.39	1.33–1.46	0.001	1.28	1.20–1.36			
GD dimension	8759	4	16	4.52	1.19	0.001	2.158	1.93–2.39				0.001	1.61	1.40–1.86
CU dimension	8759	4	16	4.59	1.32	0.001	1.75	1.58–1.94				0.350	1.08	0.92–1.28
INS dimension	8759	4	16	7.30	2.35	0.001	1.77	1.60–1.96				0.178	1.13	0.95–1.35
ADHD module	8753	0	19	1.90	2.96	0.001	1.42	1.34–1.47	0.001	1.20	1.11–1.29			
Concentration and Attention domain	8754	0	9	1.01	1.72	0.001	1.75	1.61–1.89				0.01	1.26	1.07–1.50
Impulsiveness and Activity domain	8757	0	10	0.89	1.59	0.001	1.80	1.67–1.95				0.189	1.14	0.94–1.37
ASD module	8756	0	17	0.75	1.52	0.001	1.48	1.38–1.57	0.871	0.99	0.89–1.11			
Interaction domain	8744	0	6	0.26	0.61	0.001	2.89	2.43–3.44				0.744	1.06	0.73–1.54
Language domain	8756	0	6	0.25	0.59	0.001	2.23	1.87–2.65				0.338	1.21	0.82–1.73
Flexibility domain	8758	0	5	0.24	0.59	0.001	2.72	2.31–3.19				0.531	0.91	0.66–1.24

*CPTI – SV* The Child Problematic Traits Inventory – Short Version, *GD* Grandiose/Deceitful, *CU* Callous/Unemotional, *INS* Impulsivity/Need for Stimulation, *ADHD* Attention Deficit/Hyperactivity Disorder, *ASD* Autism Spectrum Disorder

† Theoretical range and sample range are identical for all scales and subscales

## Discussion

This study mainly investigated to what extent psychopathic traits, alone and in conjunction with NDPs (i.e., ADHD and ASD), were associated with being screened positive for CD. A main finding was that the CPTI-SV GD dimension was significantly associated with CD. Moreover, the other two psychopathy dimensions (i.e., the CPTI-SV CU and INS subscales) failed to display any significant associations with screening positive for CD in the presence of co-occurring NDPs. This counters the results of many previous studies indicating an association between CU traits and CD.

As expected in a population-based non-clinical sample of youth, elevated psychopathic traits were rare, resulting in a markedly skewed distribution of scores. An overwhelming majority of the children displayed very low or zero scores for the three CPTI-SV subscales, although scores on the INS subscale were relatively higher. Unlike previous research finding an age-related increase in the GD dimension for both boys and girls (Colins et al. 2014), this study did not find this effect in the CPTI-SV dimensions. This might be because our study population was somewhat older than those in several previous studies, and that these personality traits are more developed and manifest when children reach the preadolescent phase.

We found statistically significant correlations between CD and NDPs (i.e., ADHD and ASD) as assessed with A-TAC. This was expected, given the large sample size as well as previous research findings indicating both comorbidity and symptom overlap between behavioral and neuropsychiatric conditions in youth (Blair et al. 2014; Hart et al. 1995; Rasmussen and Gillberg 2000). The finding of weak to moderately strong correlations between psychopathic traits, NDPs, and CD further supports the “ESSENCE” theory, that children with one type of neurodevelopmental problem often display other neurodevelopmental problems (Gillberg 2010). This indicates a need for preventative programs.

In our sample, there was a particularly strong correlation between the CPTI-SV INS subscale and ADHD, which may reflect content overlap (i.e., high similarity between individual items) rather than true comorbidity. That scores on both these measures were correlated with CD is unsurprising, given that impulsivity increases the risk of norm-breaking behavior and could be the common denominator between INS and ADHD. On the other hand, the weak correlation between the CPTI-SV CU subscale and ASD is surprising: we expected to find a stronger correlation, because previous research has found problems with emotional processing in ASD that are similar to CU traits (Rogers et al. 2006). The weak correlation between the CPTI-SV CU subscale and CD was also unexpected given prior research. Considering that previous research has found that CU traits specify a more severe form of CD (Edens et al. 2007; Frick et al. 2014; Frick and Nigg 2012; Frick and White 2008; Herpers et al. 2012; Viding and McCrory 2018), we expected to find a stronger correlation between the two. That the CPTI-SV GD subscale and not the INS subscale displayed the strongest correlation with CD was also surprising, given that impulsivity is a prominent feature of CD. These results add a new context to the common understanding of what aspects are most strongly related to a diagnosis of CD, and the nature of these relationships merits further study.

In previous studies, ADHD emerged as a precursor to CD (Hofvander et al. 2009). This was also found by the univariable part of this study, while in the multivariable analysis of the full model (i.e., all CPTI-SV subscales and the two A-TAC domains of ADHD and the three of ASD), only the concentration and attention part of ADHD was associated with CD. Executive functions important for attentional abilities have been found to be prominent features of ADHD in combination with CD, and also related to teacher-rated symptom severity in groups of students with ADHD only or ADHD combined with CD (Barnett et al. 2009). The association found here between the concentration and attention domain of ATAC and CD might be explained by this particularly salient attentional dysfunction found in individuals with ADHD and CD.

The multivariable analysis also suggested that, while the LPE specifier for CD in DSM-5 (largely corresponding to the CU subscale of CPTI-SV) might, as previous research has found, be useful for identifying an especially problematic subgroup of children with CD, a GD interpersonal style may be equally useful in identifying children at risk of developing CD. Indeed, it may be as strong a risk factor for CD as is ADHD. This would mean that parents should be observant of children who lie, manipulate, and exploit others significantly more than other children do, even if these children do not display other forms of antisocial behavior. The discrepancies between the present results and previous research could be because our study population was older, as older children generally tend to display higher levels of GD traits (Colins et al. 2014). It is also possible that these traits affect the development of CD at different ages, suggesting an even more complicated relationship between psychopathic traits and CD. For example, the presence of CU traits could be a stronger risk factor for CD at younger ages, or at least a more useful indicator because these traits stand out more clearly in young children. The GD interpersonal style, in contrast, might become a risk factor at a greater age, or may simply emerge later and therefore not be visible for clinical evaluation until the child has reached late childhood. Our results are also interesting with regard to the question that Salekin (2016), among others, has asked: Is there a core dimension of psychopathic traits in children and, if so, what is it? Elevated CU traits may be a root of psychopathy and expected to precede other peripheral or secondary features, but our results highlight the importance of GD as another potential core feature of psychopathy. Clearly, more research is needed in this area, but judging from the current results, it seems that clinicians interested in detecting and preventing CD may need to assess different dimensions of childhood psychopathic traits depending on the age of the child undergoing assessment, making the task even more complex.

### Strengths and Limitations

This study has several strengths, including a large twin dataset representative of the general population (Andrew et al. 2001) and the inclusion of a child psychopathy measure. However, three specific limitations affect this study. First, ASD, ADHD, and CD were derived from parent ratings alone. Not having access to either teacher reports or clinical diagnoses and professional ratings could affect the validity of the ratings. Second, the threshold for CD might be considered low. The criteria for CD (i.e., the dependent variable) are arguably over-inclusive, given that they are based on the endorsement of only two types of antisocial behavior; in contrast, clinical diagnosis requires the endorsement of at least three separate symptoms. On the other hand, previous research has demonstrated that the threshold used has high specificity (Kerekes et al. 2014), which would reduce this problem. Third, psychopathy was measured based on parent reports alone, restricting the validity of this variable. Parents might find it difficult to rate their own children; notably, a substantial part of the original CATSS population could not be included in this study because their parents had not completed the full CPTI-SV. Those completing CPTI-SV might be biased relative to those who did not, possibly reducing the generalizability of the results and weakening the conclusions.

## Conclusions

The current results lend further weight to the view that professionals working with children should broaden their scope in assessing psychopathy traits in youth with CD. Specifically, our results indicate that when considering whether a child or adolescent is at risk of developing an antisocial behavioral disorder (i.e., CD), one should look beyond the DSM-5 focus on CU traits and also examine GD traits, that is, behaviors such as lying, cheating, and manipulating others. Moreover, all three psychopathy dimensions could be used as specifiers of CD in clinical assessment, as suggested by Salekin (2016). Our results are in line with such a strategy, demonstrating that GD traits and not only CU traits are associated with CD, at least in a somewhat older study population. Finally, these results also support the notion that various symptoms of mental health problems tend to aggregate in affected individuals, likely due to common genetic factors. We therefore need to be more accurate in assessments identifying the whole spectrum of mental problems, and thus the mental health needs of assessed children, to prevent them from traveling further toward maladjustment, marginalization, and mental illness.

## Footnotes

1 Data can be obtained from the first author upon request.
